# Brain Image Segmentation in Recent Years: A Narrative Review

**DOI:** 10.3390/brainsci11081055

**Published:** 2021-08-10

**Authors:** Ali Fawzi, Anusha Achuthan, Bahari Belaton

**Affiliations:** School of Computer Sciences, Universiti Sains Malaysia, Gelugor 11800, Malaysia; alifawzi95@student.usm.my (A.F.); bahari@usm.my (B.B.)

**Keywords:** brain image segmentation, machine learning, deep learning, tumor

## Abstract

Brain image segmentation is one of the most time-consuming and challenging procedures in a clinical environment. Recently, a drastic increase in the number of brain disorders has been noted. This has indirectly led to an increased demand for automated brain segmentation solutions to assist medical experts in early diagnosis and treatment interventions. This paper aims to present a critical review of the recent trend in segmentation and classification methods for brain magnetic resonance images. Various segmentation methods ranging from simple intensity-based to high-level segmentation approaches such as machine learning, metaheuristic, deep learning, and hybridization are included in the present review. Common issues, advantages, and disadvantages of brain image segmentation methods are also discussed to provide a better understanding of the strengths and limitations of existing methods. From this review, it is found that deep learning-based and hybrid-based metaheuristic approaches are more efficient for the reliable segmentation of brain tumors. However, these methods fall behind in terms of computation and memory complexity.

## 1. Introduction

Brain imaging is important for the diagnosis of brain-related diseases such as neurological disease (Parkinson’s disease), neurodegenerative disease (Alzheimer’s syndrome), and brain tumors. According to the American Cancer Society and the National Cancer Institute Report, brain and nervous system cancer is the tenth most common cause of death for both genders. About 18,020 deaths (10,190 males and 7830 females) and 23,890 new cases (13,590 males 10,300 females) among adults were estimated due to primary cancerous brain tumors and other nervous system diseases in 2020 in the United States [1]. Therefore, early detection of brain tumors and related brain structures using effective brain imaging techniques is important where treatment can be initiated at an early stage of the brain tumor. High-quality brain images can be produced using magnetic resonance (MR) imaging, a standard non-invasive imaging key technique. MR imaging is useful for the diagnosis and treatment of brain tumors without inflicting harmful radiation on other brain structures and skull artifacts of the patients [2]. MR images are used to differentiate suspicious regions of the brain tumor from healthy brain tissue. Conventionally, location, shape, and type of brain tumors are identified visually using multimodal MR images by qualified medical doctors.

**Figure 1:**
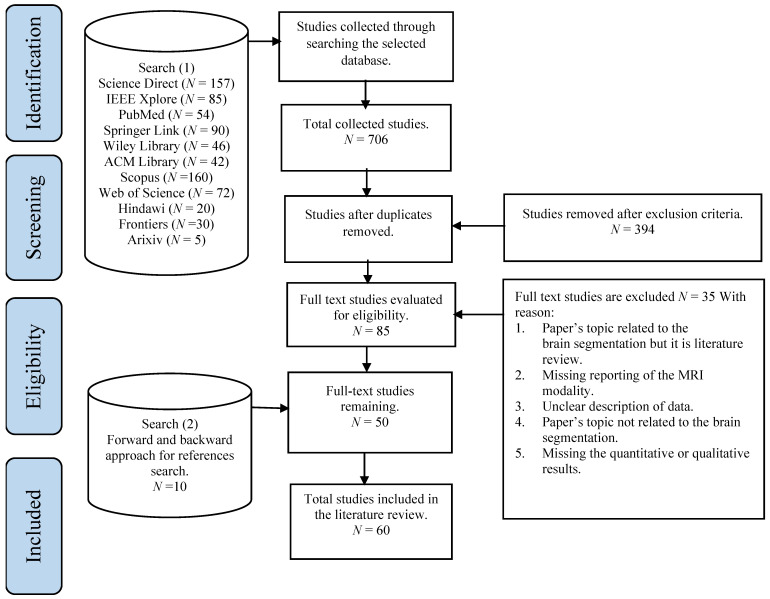
An overview of the study search and selection process according to PRISMA guidelines [4].

Accurate and consistent segmentation of target brain regions or tumors from the surrounding tissues using the MR images is crucial for clinical evaluation of disease progression, surgical planning, post-surgical matching, and radiation therapy outcomes. Since the manual segmentation of the brain tumors and related small brain structures is laborious and time-consuming, many automated solutions have been explored and presented thus far. Generally, the automated brain segmentation methods using MR images can be classified into three main theoretical categories (Figure 1), namely, (i) intensity-based, (ii) machine learning, and (iii) hybrid, which are explained in this paper. Section 2 discusses the search methodologies used in this review. Next, this paper discusses the typical segmentation approaches and the respective challenges in Section 3. The conclusion of the present critical review is presented in Section 4.

## 2. Search Strategy and Selection Criteria

The present review aims to summarize information and identify problems from relevant research articles that utilized computer vision techniques in the context of automated brain medical imaging over the last five years. The inclusion and exclusion criteria were applied to conference papers and journal articles published on brain medical imaging in the chosen databases from 2016 until 2021. Studies that were not written in English, were duplicative, out of the study period, and did not have the full text available were excluded.

The studies were collected using the search keywords on 11 selected databases (i.e., Scopus, Web of Knowledge, Science Direct, IEEE Xplore, Springer, Frontiers, Wiley Online Library, Arixiv, ACM Digital Library, Hindawi). These databases offered comprehensive literature regarding brain image segmentation approaches and are highly appropriate. First, a search was conducted on the basis of the following keywords/terms: (delineation OR segmentation OR contouring) AND (brain tumor OR neoplasia OR brain tissues OR brain anatomical structure)) AND (ALL(“automatic delineation” OR “automatic segmentation” OR “automatic contouring” OR “semi-automatic delineation” OR “semi-automatic segmentation” OR “semi-automatic contouring”)) AND (brain tumor OR neoplasia OR brain tissues OR brain anatomical structure)) AND intensity-based methods—((delineation OR segmentation OR contouring) AND (“thresholding” OR “region” OR “Otsu” “level set” OR “active counter”) (brain tumor OR neoplasia OR brain tissues OR brain anatomical structure) AND (ALL(“automatic delineation” OR “automatic segmentation” OR “automatic contouring” OR “semi-automatic delineation” OR “semi-automatic segmentation” OR “semi-automatic contouring”)) AND (PUBYEAR > 2015) AND (“MRI”))), machine learning methods ((delineation OR segmentation OR contouring) AND (“clustering” OR “classification” OR “deep learning” OR SVM OR ANN OR K-means OR FCM OR FCN OR CNN OR convolution OR UNet OR U-Net) AND (brain tumor OR Neoplasia OR brain tissues OR anatomical structure) AND (ALL(“automatic delineation” OR “automatic segmentation” OR “automatic contouring” OR “semi-automatic delineation” OR “semi-automatic segmentation” OR “semi-automatic contouring”) AND (PUBYEAR > 2015) AND (“MRI”))), and so on, as well as for the hybrid method and its subcategories.

In the beginning, 761 publications were retrieved by searching the selected databases. An additional 15 publications were identified through cross-referencing. Following duplicate publications removal, the remaining 459 publications were evaluated via exclusion criteria. Based on screening the title and abstract, 394 publications were excluded. A total of 85 full-text studies were evaluated for eligibility, and 50 papers were included in this review. Then, an additional search was conducted on all selected publications by using the backward and forward approach for the references search method introduced by Webster and Watson [3]. Through the backward search, the citations of each publication were assessed to obtain further publications to be included in the review. Through the forward search, for example, the references were obtained using Google Scholar were used to obtain further relevant studies. The results reported 10 additional publications. Overall, a total of 60 publications were selected. Figure 1 illustrates the search strategy with the publications’ selection methods.

**Figure 2:**
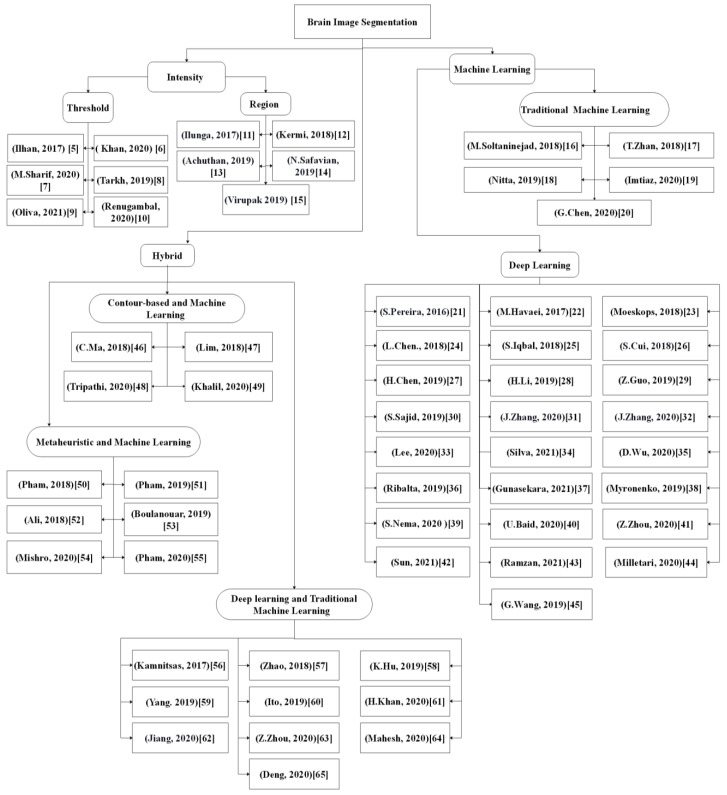
An overview of brain segmentation approaches [5,6,7,8,9,10,11,12,13,14,15,16,17,18,19,20,21,22,23,24,25,26,27,28,29,30,31,32,33,34,35,36,37,38,39,40,41,42,43,44,45,46,47,48,49,50,51,52,53,54,55,56,57,58,59,60,61,62,63,64,65].

Figure 2 shows the taxonomy of reviewed research papers published in the last 5 years. It was noted that recent publications predominantly applied deep learning and hybridized metaheuristic-based methods for brain image segmentation.

## 3. Brain Segmentation Approaches

### 3.1. Intensity-Based Approaches

The intensity-based methods for brain segmentation function in the spatial domain and depend on the pixel value, which can be further classified into a thresholding and region-based approach.

#### 3.1.1. Thresholding

The thresholding approach is one of the conventional and the easiest image segmentation methods where the regions of the image are categorized by measuring their intensities and compared with one or more intensity thresholds. For instance, Otsu’s method enables the determination of the global threshold optimal value to distinguish the target object from the image background. In a previous study, Otsu’s thresholding approach was coupled with morphological operations to detect brain tumors using MR images [5]. Another study by Khan et al. [6] presented a grade-wise brain tumor identification method where segmentation of the tumor was conducted first through the threshold approach. Then, a logical formula was employed to extract the desired tumor region. Moreover, feature set parameters, such as the angle, area, density, solidity, size, center of mass, and perimeter, were extracted from the tumor region. The extracted features were then analyzed using the partial tree (PART) algorithm to grade the brain tumor. However, the thresholding approach is sensitive to noise and intensity non-homogeneity, which limits its application for the entire tumor region. To overcome the limitation, statistical optimization of the threshold method was reported by Sharif et al. [7], where particle swarm optimization (PSO) was employed to achieve the maximum class variance between the tumor regions and healthy brain tissues. Then, hand-crafted local binary patterns (LBP) and deep (fine-tuned capsule network) features of segmented images were extracted, and the best features were selected using a genetic algorithm (GA). Finally, an artificial neural network (ANN), a support vector machine (SVM), and an ensemble of linear discriminant analysis (LDA) were utilized to classify the tumor grades.

The above-mentioned methods have several limitations such as (i) low convergence rate and an insufficient local and global search and (ii) the optimization being trapped into a local minimum that results in low segmentation accuracy. To improve the local and global search of the multi-level thresholding approach, a new metaheuristic approach of the differential evolution (DE) technique, which was termed as adaptive differential evolution with Lévy distribution (ALDE), was introduced by Tarkhaneh and Shen [8] for brain tissue segmentation. The proposed approach was adopted to resolve the multi-level threshold issue and achieve optimal results by preventing a local minimum through the establishment of a balance between exploration and exploitation coupled with the convergence rate boost. However, some of the tested images in this model did not segment properly, which could be attributed to the limitation of the thresholding approach that does not consider the spatial information of images, resulting in insensitivity towards different levels of noise and intensity.

Oliva et al. [9] proposed an adaptive differential evolution and linear population size reduction (LSHADE) metaheuristic algorithm to determine the optimal threshold value by employing the minimum cross-entropy as a fitness function for the segmentation of brain tissue from MR images.

In a different study [10], Renugambal et al. proposed a new hybridization approach based on the Otsu and new hybrid water cycle and moth-flame optimization algorithm (WCMFO) for the brain tissue segmentation. The new WCMFO algorithm was proposed to determine the optimal values for Otsu’s objective functions on various axial T2 modalities of MR brain images. However, the model cannot convert several parameters, including the water cycle and moth-flame algorithms.

#### 3.1.2. Region-Based

The region-based approach enables the extraction of a connected region of an image by following pre-defined conditions such as pixels’/voxels’ information with matching intensities. This approach is performed in three steps: (i) selection of an initial seed point, (ii) locating the points in objects or regions, and (iii) selection of points connected to the initial point with similar intensity values. Recently, several studies applied a region-based approach for brain tissue segmentation [11,12,13,14,15]. 

A semi-automatic approach, which consists of a localized active contour integrated with a background intensity compensation, termed LACM-BIC for tumor region segmentation, was presented by Ilunga-Mbuyamba et al. [11]. The T1 contrast and T2 MR images were fused and used to segment the tumors. An automated initialization of the initial contour in the LACM-BIC method was conducted using the k-means algorithm accompanied by a hierarchical centroid shape descriptor. This method chose the best initialization number of the cluster, k, for the k-means algorithm, in which wrong selection of the initialization may lead to unwanted regions in the segmentation. Hence, the contour may be trapped into the wrong local minimum.

In another region-based study, a 3D-MR image brain symmetry analysis for tumor segmentation was reported by Kermi et al. [12]. Specifically, the fast-unsupervised bounding box (FBB) and geodesic level-set methods were used. The FBB algorithm was adopted to locate initial tumor voxels and to manage intensity variations among different MR images without the use of a training dataset. Subsequently, the region growing method was combined with a 3D level set method to acquire the final tumor region. The drawbacks of this method include the inability to avoid noise and non-uniform intensity besides being limited to tumor segmentation. 

In a more recent study, Achuthan and Rajeswari [13] presented an automated point set registration approach to establish a prior knowledge model with a lower data intensiveness for hippocampus segmentation. In comparison to the usage of the entire 3D volume as used in the atlas-based methods, this study utilized a collection of representative points on the boundary of the hippocampus. The prior model was created and integrated into a level set model to perform hippocampus delineation. Nevertheless, some parameters are required to be specified experimentally, and it is a subjective task that depends on the target image properties.

Another approach for hippocampus segmentation of MR images using an automated level set method has been proposed by Safavian et al. [14]. First, prior knowledge was obtained from an affine registration with a non-linear registration stage. Then, this information was locally integrated into an innovative level-set framework using a binary weighting map. The image gradient information adaptively utilized both local and global region information of the corresponding image. However, manual setting of parameters is required, which is very subjective and depends on the target image properties. 

In a different study, Virupakshappa and Amarapur [15] presented a modified level set segmentation method for brain tumor segmentation that provides an automatic initialization point as indicated by the maximum pixel point that serves as the initial contour. The maximum pixel was determined from the histogram, and an automatic segmentation was performed using an anisotropic diffusion filter instead of the Gaussian filter. The utilization of the anisotropic diffusion filter enhanced the local edges by detecting discontinuity within the local edge. Boundaries formed as a result of noise were removed completely, and the contours of the object were also improved. However, the manual setting of the initial contour of the level set is required to be performed based on the maximum pixel point, which is very subjective and depends on the intensity non-homogeneity.

### 3.2. Machine Learning

Another category of brain image segmentation approaches is traditional machine learning, comprising clustering and classification and deep learning approaches. The sections below detail these approaches.

#### 3.2.1. Traditional Machine Learning

The clustering and classification approaches, being the traditional machine learning methods, are motivated by multidimensional feature space that may be obtained from different MR modalities. Classifiers are trained using a feature space that is created by combining different intensity and textural-based features representing the known classes. Then, a class prediction that the target structure belongs to is performed by assigning a class label, which is most similar to the target structure’s feature space. Meanwhile, clustering methods are unsupervised pixel-based methods that segregate unlabeled images into clusters of pixels that have similar features without utilizing training images. Some of the machine learning-based methods were applied for brain tissue segmentation in recent studies [16,17,18,19,20]. A 3D super-voxel learning method for brain tumor segmentation was proposed by Soltaninejad et al. [16]. In the study, the MR images were partitioned into the equivalent size of patches with similar intensity ranges based on the simple linear iterative clustering (SLIC) algorithm. Super-voxel clusters were formed by combining information from the MR multimodal images using a distance metric. For each super-voxel cluster, a set of texton descriptors along with the first-order static features were extracted from different MR modalities. The features were then used to train a random forest (RF) classifier to classify each super-voxel into a core tumor, edema, or healthy tissue. This approach was found to effectively combine the unsupervised SLIC algorithm for initial tumor region localization and the supervised RF method for tumor classification. The approach enabled the classification of located regions into sub-regions in a unified system and resulted in promising segmentation findings. However, this approach limits the segmentation of complex structural boundaries such as the smaller tumor cores as the super-voxel may include voxels from various tissue types.

In another study, a semi-supervised method based on a co-training technique with clinical and spatial constraints for the extraction of the glioma region, namely whole tumor (WT), tumor core (TC) and enhancing tumor (ET) from multi-sequence MR images, was assessed by Zhan et al. [17]. Firstly, the labeled brain MR image was used for both SVM and sparse representation classification (SRC) classifiers training. This allows the extraction of high confidence data as pseudo-labeled samples from the test samples. The pseudo-labeled samples that resulted from each classifier were then added to the training sets of the other side of the classifier to re-train the corresponding classifier. The process was iteratively repeated until the results of classification remained stable. Finally, a super-pixel graph was plotted on the post-contrast T1 image to generate spatial and clinical constraints to remove false-positive and interference of noise. This classification method provides generalization fitting using a limited training set. However, it has a drawback where prior clinical knowledge is required to refine the segmentation results by manually correcting the pixel labeling, which is subjective as it depends on the user’s expertise.

Meanwhile, Nitta et al. [18] investigated an approach for brain tissue segmentation using a modified k-means clustering algorithm. The researchers proposed a selection of 16 high probabilities of dominant grey-level pixels as initial centroids to resolve the issue of the arbitrary selection of initial centroids in the standard k-means algorithm. The proposed approach is sensitive to noise and has a non-uniform intensity distribution.

In a recent study by Imtiaz et al. [19], a tumor segmentation approach based on super-pixel features extracted from 3D planes of MR images (FLAIR, T1c, and T2 modalities) was evaluated. Several statistical and Gabor textural features were extracted from each super-pixel of the three planes to avoid imbalanced planar data and mislabeling of pixel issues in a plane. Based on feature effectiveness, feature selection was performed using histogram consistency analysis and local descriptor pattern analysis. The feature vector for each super-pixel was then subjected to extremely randomized trees (ERT) for binary classification. Then, the voting algorithm was used to assign a class label (tumor or non-tumor) for each pixel in all three planes. The benefits of this approach include fast computation and high robustness to the scale-invariant and rotational changes. However, this approach is sensitive to noise and distortions, in addition to leading to the extraction of redundant features at different scales. 

In a different study by Chen et al. [20], a hybrid two-stage framework of cascaded RF and a dense conditional random field (CRF) was evaluated for intra-tumor segmentation. Firstly, the appearance features (statistical intensity and template-based) and contextual features (Gaussian mixture model-based lesion tissue probability maps) were extracted and used to train the initial RF classifier. The predicted probability map obtained by the RF classifier was used as the prior input into a dense CRF model for further segmentation improvement. Then, the results of the dense CRF model were used as the contextual information to train a cascade of RFs by the hierarchy in combination with template-based asymmetrical and original statistical features. The authors proposed a multi-layer optimization architecture as the post-processing step to further increase the efficiency of RF. The step is easy to implement and can be effectively incorporated into the local appearance and global contextual features, which can improve the segmentation outcome. The limitation of this framework includes the evaluation was performed using a small dataset, and a post-processing step is required to fine-tune the extracted tumor regions.

#### 3.2.2. Deep Learning

Recently, the deep learning-based method has attracted much research interest due to its excellent performance and ability to automatically capture adaptive features, which outperform manually created features. Moreover, these features were learned in an increasing feature complexity trend, which results in more robust feature learning. During the last few years, more studies have been designed using a combination of the deep learning-based method and the new brain tumor segmentation method. Most of the studies utilized convolutional neural networks due to their effectiveness in detecting patterns in an image, specifically the MR images, with promising results reported. To date, the deep learning-based segmentation was performed using 2D, 2.5D, or 3D MR images, which is elaborated in the following sections.

##### Deep Learning-Based Methods Using 2D Images

Deep learning using 2D images requires brain image slices or extracted 2D patches from 3D images as an input for the 2D convolutional kernel. Several studies [21,22,23,24,25,26,27,28,29,30,31,32,33,34,35,36,37] have been published on the deep learning-based method using 2D images. Sergio Pereira et al. [21] introduced cascade layers using small 3*3 convolutions kernels to reduce overfitting. The study enabled the segmentation of the image into four regions, namely (i) necrosis, (ii) enhancing tumor, (iii) edema, and (iv) normal tissue. Two convolutional neural network (CNN) architectures were trained and used in the proposed work to extract the feature maps, which were low-grade glioma and high-grade glioma. The use of small kernels led to a deeper architecture design, which reduced the number of weights in the network and significantly affected overfitting. However, for the initial phase, the user has to manually identify the glioma grade where prior medical knowledge is required, which is one of the limitations. Additionally, the tissue segmentation was performed as a patch-based task in the study where the local dependency of labels during pixel classification was ignored. Another drawback of the proposed method is the poor segmentation of tumor core regions in the BRATS 2015 Challenge dataset. 

Similarly, the application of another novel Cascade CNN model for fully automatic brain tumor segmentation was reported by Havaei et al. [22]. Cascade architecture of 2D CNN was used in the study to preserve local dependency of labels during pixel classification and extract local and global contextual features which deal with imbalanced tumor labels. However, the model suffers from two drawbacks: (i) poor segmentation between the enhanced and core regions of the brain tumor inferior to the complete tumor and (ii) only the local dependency of the labeled samples was considered, with the appearance and spatial consistency neglected when applied on 3D images.

Moeskops et al. [23] presented an automatic approach based on a multi-scale CNN for segmenting white matter hyperintensities of presumed vascular origin (WMH) (basal ganglia and thalami, brain stem, cortical grey matter, white matter, cerebellum, WMH, peripheral cerebrospinal fluid, and lateral ventricular cerebrospinal fluid) from MRI modalities (T1, T2, FLAIR, and T1inversion recovery). The proposed multi-scale CNN model was claimed to be the first modern MRI segmentation method that applies CNN for additional WMH segmentation. Furthermore, the model was assessed in two large MRI datasets of older patients that were affected by motion artifacts and varying degrees of brain abnormalities.

Another study by Chen et al. [24] proposed a 2D novel method based on a CNN architecture identified as Dense-Res-Inception Net (DRINet) for multi-class brain tumor segmentation. The DRINet consisted of three blocks, namely, (i) convolutional, (ii) deconvolutional, and (iii) unpooling blocks. The convolutional block carried out dense connections and was used to alleviate the effect of vanishing gradients. Meanwhile, the deconvolutional block carries out the residual inception modules to aggregate feature maps from different branches. The unpooling block was used for the aggregation of different sampled feature maps. The use of this method resulted in accurate findings on segmenting complex, challenge, multivariate domains (tumor and cerebrospinal fluid (CSF)), and multi-organ segmentation on abdominal CT images. Nevertheless, the DRINet approach has a complex network structure that requires millions of parameters (i.e., billions of connections between neurons and millions of weights), which could lead to a difficult training phase, and testing can be slower depending on the ground truth label requirements. 

Iqbal et al. [25] proposed three different improved network architectures for intra-tumor segmentation, which were an extended version of SegNet (deep convolution encoder-decoder architecture), as follows: (i) Interpolated Network, (ii) SkipNet, and (iii) SE-Net. All three structures consisted of decoder/encoder architecture, and four sub-blocks were used in each phase. A batch of normalization layers was added next to each convolution to avoid the disappearance or explosion of convolutional gradients and to maintain the stability of the training phase. The advantage of this approach includes the use of simple network structures as an intermediate convolutional map along with interpolation methods to produce a quick model with a smaller memory space. However, the method has a limitation where the segmentation performance could be affected if the model is trained with limited ground truth samples. 

In the same year, Cui et al. [26] reported on a hybridized cascade of a deep convolution neural network (DCNN) architecture that can segment 2D brain images automatically in two major steps. Firstly, the tumor region was localized immediately using the pixel-wise fully convolution network (FCN) from the MR images. Then, the patch-wise CNN with smaller kernels and deeper architecture was adopted for further classification of the localized tumor region into multiple sub-regions. This approach alleviates the imbalanced data issue using a hybrid CNN. However, the approach is time-consuming during model training, and inference is required for operating the image patches. 

In a different approach, Chen et al. [27] presented a combination of prior knowledge and a DCNN to enrich the extracted features of DCNN for brain tumor sub-compartment identification. This model requires an analysis of a left-right similarity mask (LRSM) in the constructed feature space and uses LRSM as the location weight of the DCNN features. These features were then used to train the model to determine the asymmetrical location information of the input images via a similarity metric. This approach was found to provide about 3.6% of dice similarity coefficient (DSC) improvement of complete tumor segmentation over the conventional DCNN. The advantage of the proposed method includes the ability to combine the symmetric masks in several layers of DCNN to assign location weight for the extracted features. However, the method could not differentiate between the tumor core regions and the enhanced tumor region as the LRSM mask can reflect a complete tumor situation. 

Li et al. [28] presented an automatic approach based on the improved version of U-Net for multiclass brain tumor segmentation from 2D MR image slices. Firstly, the up-skip connection between the encoding and the decoding elements was proposed to further enhance the information flow and the network connectivity. Then, in each block, an inception module was implemented to assist the network in learning richer representations. Nevertheless, the model suffers from poor segmentation of enhancing tumor region as the whole brain slices were used for model training. This led to a data imbalance issue due to a small number of pixels that belong to enhance tumor and core regions inferior to other brain tissue.

Another approach was reported by Guo et al. [29], where a supervised multimodal image analysis was performed with three cross-modality of fusion level strategies, which were feature learning, classification, and decision making. The three fusion strategies were implemented and tested in three different patches-based CNNs with corresponding variations in the network structures. Four modalities of imaging (CT, PET, T1, and T2) were used as fused inputs for brain tumor segmentation. Comparison between the single model and multimodality showed that the CNN-based fusion network performed better on PET, CT, and T2 modalities. This approach provides methodological guidelines for designing and applying multimodal image analysis fusion strategies through different implementations of CNN architecture. However, this approach is limited for complete tumor detection. Another limitation is the dramatic decrease in the segmentation performance within the misaligned regions based on the number of affected modalities and severity of the misalignment.

An automated hybrid DCNN model for brain tumor segmentation was presented by Sajid et al. [30] for different modalities of MR. This model extracted 27 × 27 sized patches from four axial MR modalities to consider both spatial and contextual knowledge for predicting segmentation labels of pixels. The proposed hybrid DCNN model combined the output feature maps of two- and three-CNN paths. The model successfully addressed local dependencies between the output labels, which was the major drawback of the two- and three-CNN paths. By integrating the two- and three-CNN networks, an increase in the effect of neighboring pixels was noted, and the output was recognized based on the local and contextual features. Morphological operations were used to further enhance the segmentation performance by eliminating minor false positives along the edges of the expected outputs. The proposed model segmented the core and enhanced tumor regions better compared to the complete tumor regions. This could be attributed to the fuzzy boundaries of edema that limit the detection of the whole tumor region compared to other regions. However, this approach has a limitation where a large amount of training data and parameters are required for model training. 

In addition to the various methods proposed, Zhang et al. [31] presented a residual U-Net and attention mechanism in a unified architecture named AGResU-Net for patch-wise brain tumor segmentation. Attention gate units were added into the up-skip connection of the U-Net structure to highlight the important feature details along with disambiguates in noise and irrelevant feature responses. The AGResU-Net was found to enhance feature learning by extracting important semantic features focusing on the details of small-scale brain tumor sub-regions, which improves the segmentation performance of the brain tumors. Nevertheless, the AGResU-Net model has a drawback, where an amount of contextual information and local details among different intra-slices were not included due to modeling based on 2D U-Net.

In the same year, Zhang et al. [32] proposed another new method using attention residual U-Net (AResU-Net) for end-to-end 2D brain tumor segmentation. The AResU-Net embedded a series of attention and residual units among corresponding down-sampling and up-sampling processes. The system simultaneously improved the local responses of down-sampling and the recovery effects of the up-sampling process. However, the model neglects contextual and local details of different intra-slices due to modeling based on 2D slices.

Recently, an innovative brain tissue segmentation method from MR images was proposed by Lee et al. [33], where a patch-wise U-net architecture was used to divide the MR image slices into non-overlapping patches. Corresponding patches of ground truth were incorporated into the U-net model, and input patches were predicted individually. The model was found to retain the local spatial information better compared to the conventional U-Net model. The design successfully fixed the drawback, specifically the limited memory problem, which was caused by multiple down and upsampling stages. The memory problem was attributed to the storage of parameter values at each stage and difficulty in maintaining local details as the entire image is incorporated into the network. Although the memory problem was resolved using the proposed model, computational complexity was higher in the training phase. 

In another study, Silva et al. [34] proposed a three-stage cascade FCN architecture based on the deep layer aggregation technique to gather further spatial and semantic information for intra-tumor segmentation. The output features of one FCN are directly fed to the next layer for extending the feature hierarchy over different depths for better segmentation refinement. However, the model requires high computational resources and post-processing to refine the extracted tumor regions. 

In addition to the various proposed methods, Wu et al. [35] suggested a multifeatures refinement and aggregation network (termed MRANet) based on CNN for end-to-end brain tumor segmentation. The model fully utilized the hierarchical features by adopting the feature fusion concept at several levels, which extracts low-level, mid-level, and high-level features by sampling similar hierarchical features of encoder and decoder. These features were then aggregated and re-extracted for better segmentation refinement.

Ribalta Lorenzo et al. [36] proposed a deep learning method for brain tumor delineation from the FLAIR modality of MR using the fully convolution neural network (FCNN) inspired by U-Net. The authors trained the model on 256 × 256 patches extracted from the intra-tumor regions that belong to only positive (tumorous) full-sized FLAIR MR image sequences. Firstly, data augmentation methods were used to expand the dataset and achieve a robust algorithm against the heterogeneity of small training datasets. Subsequently, the FCNN was trained using the DSC to maximize the model training to improve the quality of the segmentation. The proposed FCNN model was claimed to be the best modern FLAIR MR image segmentation method that applied hand-crafted features and was classified using extreme random trees. This model offers controllable training time and instant robust segmentation using the FCNN that was trained on heterogeneous and imbalanced datasets. Nevertheless, this model exhibited potential drawbacks caused by the rapid data augmentation process, as the unnatural increasing number of training patches resulted in a reduction in overall average data accuracy.

Gunasekara et al. [37] proposed cascaded algorithms for glioma and meningioma brain tumor segmentation and classification. Firstly, CNN was implemented to classify meningioma and glioma regions. Then, the classified images were fed to R-CNN to localize the tumor regions of interest, which was accompanied by active contouring to delineate the exact tumor boundary. Finally, the Chan–Vese level set model was used to segment the target tumor boundary.

##### Deep Learning-Based Methods Using 3D Images

The second category of deep learning-based tumor segmentation approaches uses 3D MR images for segmentation to overcome the limitation of neglecting contextual information in 2D CNN. Several studies [38,39,40,41,42,43] have reported the approaches under this sub-class. 

The intra-tumor region segmentation method from 3D MR images based on the asymmetric encoder-decoder network was presented by Myronenko [38]. The researchers adopted CNN’s encoder-decoder structure with an asymmetrical large encoder to extract deep features and reconstruct the dense segmentation masks using a decoder. To tackle the issue of a small training dataset, a variational auto-encoder was added to the endpoint of the encoder, and the input image was reconstructed together with the segmentation to regularize the shared encoder at the inference time. This model enables accurate intra-tumor segmentation based on the unsupervised feature learning method with a lower requirement for ground truth labels and without the post-processing step. However, the proposed method requires high computational resources to accelerate tumor annotation in MR images.

To decrease the dependency on the ground truth images during the training stage, Nema et al. [39] proposed a RescueNet approach for multi-class brain tumor segmentation utilizing both residual and mirroring principles. Different training was performed to segment whole, core, and enhancing tumors using three different networks. The proposed RescueNet approach was trained based on the unpaired generative adversarial network (GAN) method, which was utilized to enrich data for the training stage with better segmentation results obtained using a larger amount of testing data. Finally, a scale-invariant algorithm was suggested as a post-processing stage to improve the segmentation accuracy. The pros of this approach include robustness to the appearance variations in brain tumors, the minimum requirement of labeled datasets for model training, and that the model is 10% trained and 90% tested. However, this approach requires a post-processing step for further segmentation refinement. 

In a more recent study by Baid et al. [40], an effective weighted patch extraction was combined with a new 3D U-Net architecture for a fully automatic brain tumor segmentation. The authors proposed a weighted patch-based segmentation approach to address the imbalance of class among tumor and non-tumorous patches. The 3D weighted patch-based method and a unique number of feature maps were designed to train the architecture, which enables the accurate segmentation of intra-tumor structures. Finally, a 3D connected component analysis was used as the post-processing method to improve the accuracy of the tumor delineation. However, this approach failed to segment some of the tumor parts with a small necrotic tumor cavity from the MR images due to a large variance in the training and validation dataset features. This can be resolved by increasing the number of training data to overcome the inter-patient variations. 

To address the two main challenges, namely, exploding and vanishing gradients affecting the traditional DCNNs performance, Zhou et al. [41] proposed a novel three-phase framework for automatic brain tumor segmentation of the 3D MR images. Firstly, a dense three-dimensional networking architecture was adopted to construct the features to be re-used. Secondly, 3D atrous convolutional layers were used to design a new feature pyramid module, which was added to the backbone end to fuse the multiscale contexts. Finally, for further training promotion, a supervision 3D deep mechanism was equipped to enhance the network convergence by adding auxiliary classifiers to alleviate the problem of exploding and vanishing gradients by utilizing dense connectivity. Overall, this framework is considered a complete architecture without additional post-processing stages. Furthermore, simple implementation and the use of adjustable parameters are the main advantages of this framework. However, the segmentation of cores and enhancing tumors are inferior compared to the complete tumor, which requires considerable improvement.

In another study, Sun et al. [42] presented a multipath way 3D FCN architecture for brain tumor segmentation. The model extracts different receptive fields of feature maps from multi-modal MR images using the 3D dilated convolution in each pathway and fuses these features spatially using skip connections. This model helps FCN architectures to better locate the boundaries of tumor regions. However, the model requires a post-processing step, as direct connections between high- and low-level features will lead to unpredictable consequences and the semantic gap between the encoder and decoder.

An effective mapping from MR volumes to voxel-level brain tissue segments was proposed by Ramzan et al. [43]. A 3D CNN, which utilized the concept of residual learning, skip connections, and dilated convolutions, was applied in the study. Dilated convolutions were utilized to decrease the computational cost by computing spatial features with a high resolution. However, the space complexity of this model was higher as dilated convolution was used, and down-sampling of input volumes was neglected, which led to an increase in the number of parameters and kernels by a certain factor.

##### Deep Learning-Based Methods Using 2.5D Images

Although 3D deep neural network (DNN)-based segmentation can better exploit 3D features of 3D MR image information data, this approach has limitations related to network intensiveness and memory consumption. Therefore, another category of 2.5D DNN was researched. In comparison to the 2D and 3D DNN, 2.5 DNN has inter-slice characteristics and lower memory demand.

An automated 2.5D patch-wise Hough-CNN model based on a voting strategy for localizing and segmenting brain anatomies of interest (26 regions of the basal ganglia and the midbrain) was presented by F. Milletari et al. [44] for different modalities of MRI and ultrasound slices. The patch-based voting strategy was designed and integrated into the Hough-CNN model to localize and segment brain structures that are corrupted by artifacts or are partially visible. 

To overcome network complexity and memory consumption of the 3D based-segmentation methods, Wang et al. [45] suggested a cascade of 2.5D CNN voxel-wise architecture for sequential segmentation of brain tumors from MR images. The task of multiclass segmentation was largely divided into a sequence of binary hierarchical tasks to segment complete, core, and enhancing tumors for better utilization of hierarchical features of brain tumor structures. The resultant segments were then used as a crisp mask to identify tumor cores and enhancing tumors, which could lead to anatomical constraints during the final segmentation. The predicted tumor core was constrained to be within the whole tumor, while the enhancing tumor region was within the core tumor region. Additionally, the test-time augmentation technique was used to obtain structure-wise and voxel-wise uncertainty estimation of the segmentation results. Finally, a CRF was proposed as the post-processing stage to smoothen the segmentation results. A robust segmentation resulted in a balanced property of memory consumption, model complexity, and multi-view fusion. However, the method has two main limitations: (i) it is highly dependent on the voxel-wise annotations technique and (ii) time-consuming for large datasets. Additionally, this approach requires post-processing for segmentation tuning. The advantages and disadvantages of all of the discussed segmentation approaches are summarized in Table 1.

### 3.3. Hybrid Segmentation Approaches

Hybrid segmentation is the fourth category of brain image segmentation, which includes the integration of different methods to improve the segmentation performance and achieve the segmentation objectives. Therefore, hybrid approaches refer to the combination of two or more related methods by utilizing their advantages to achieve high segmentation accuracy. In general, hybrid-based approaches perform well, possess better designs, have shorter computational time, and have adaptive modulations towards the target task in comparison to other segmentation approaches. Hybrid segmentation can be divided into three sub-categories, namely, (i) contour-based and machine learning, (ii) metaheuristic, and machine learning and (iii) deep learning and machine learning. Each sub-category contains several approaches that aim to segment the required MR image.

#### 3.3.1. Contour-Based and Machine Learning

The combination of the contour-based and machine learning approach can improve initialization parameters, perform further spatial constraints, direct the evolution of intensity-based pipelines, and enhance data mining algorithms by refining the process. There are several previous studies [46,47,48,49] that were conducted based on this sub-category.

Ma et al. [46] hybridized concatenated and connected random forests (ccRFs) and multi patch active contour (mpAC) methods to automate the segmentation of glioma structures from volumetric multimodal MR images and impose a contour evolution on the voxel classification, which was considered as the local dependency of labels. The ccRFs were used to represent the adaptive features iteratively and efficiently to handle data imbalance issues by exploring both local and contextual information from multimodal images. Meanwhile, the mpAC technique was used for the final segmentation of the initially inferred tumor structure from the voxel classification of the ccRFs model. Although the proposed method resulted in promising findings, there are some drawbacks. Firstly, the hybridized approach highly depends on the labeled training data. Secondly, the use of multiple imaging modalities for model training on a specific feature of learning kernels and aggregation of feature maps by the max-out process is not optimal for the aggregation of imaging modalities. 

In another hybridization study, Lim and Mandava [47] proposed a semi-automatic method that incorporated both prior knowledge and image statistics in three major phases for the detection of brain abnormalities in the MR image. For the first phase, a user was permitted to determine the regions of interest using a modified random walks algorithm to perform initial segmentation and produce a feature map from each image. Then, the feature maps were incorporated into the image information and combined using the weighted averaging method. Finally, information-theoretic rough sets (ITRS) were used for the post-processing phase to locate the ambiguous boundary regions between the tumor and its background. However, the user-based interaction approach requires users to place seeds manually to distinguish between the objects and backgrounds. The inappropriate initialization of the seed can produce poor and inaccurate results. Moreover, the proposed model was only tested using limited real brain images.

Recently, Tripathi et al. [48] proposed an integrated Otsu k-means method for tumor components segmentation. This method integrated Otsu thresholding and k-means clustering to generate tumors using T2-W and FLAIR image modalities. Although this model addressed the data limitation problem, it is highly influenced by noise.

Another recent work by Khalil et al. [49] adapted the dragonfly algorithm (DA) to perform a clustering-based contouring approach for brain tumor segmentation. First, the two-step DA-based clustering was used to extract tumor edge as initial tumor contour for the MR image sequence. Instead of using a random initial position in DA, k-means was employed to identify the initial swarm centroids. Finally, the level set model was used to extract the tumor region from all volume slices. However, the usage of k-means to determine the initial centroids for DA may lead to non-stable performance because k-means is known to suffer from (i) dependency on initialization and (ii) the tendency to terminate in local optima.

#### 3.3.2. Metaheuristic and Machine Learning

The combination of metaheuristic and machine learning methods is the second sub-category of the hybridization method that can be used to optimize the separation characteristics of the machine learning method in segmented images. Additionally, this type of hybridized approach is generally used to solve or reduce the major drawbacks of machine learning segmentation methods, such as the possibility of being trapped in a local minimum and sensitivity to noise. Several studies [50,51,52,53,54,55] have employed a combination of metaheuristic and machine learning methods.

A new hybridization method for brain tissue segmentation, which is a combination of metaheuristic particle swarm optimization (PSO) method and kernelized fuzzy entropy clustering with Baize correction method and spatial information (PSO-KFECSB), was introduced by Pham et al. [50]. The approach was developed to partially overcome clustering-based segmentation problems such as (i) intensity non-uniformity (INU) artifact and sensitivity to noise and (ii) dependency on the initial clustering centroids and being trapped in local minima. However, the performance of this approach decreased with the co-existence of high noise levels and INU artifacts in the MR image data. Moreover, only one KFECSB criterion was used to direct the solution search process where the global optimum of standards may not be optimum for segmentation. The issue was solved as reported in a different study by the same group of researchers, Pham et al. [51]. A multi-objective optimization strategy was carried out to exploit the strengths of other criteria to enhance the trade-off property between preserving image details and restraining noise for image segmentation. A modified multi-objective particle swarm optimization (MOPSO) approach was proposed to optimize both objective functions of fuzzy c-means (FCM) and a region-based active contour method simultaneously to solve major drawbacks of this hybrid segmentation approach for segmenting brain tissue. This approach aimed to achieve compactness and separation by optimizing the separation between the clusters/regions from each other and consider both bias correction and spatial information in the objective functions to reduce noise effects and intensity non-uniformity artifacts. Nevertheless, this approach requires high computational time to specify the two-scale parameters (ρ, ζ), where ρ is the level of intensity inhomogeneity, and ζ is the level of noise. These parameters control the influence of global and local fitting energy force that is subjective and highly dependent on the degree of noise and INU artifact of the input images.

In another study, a hybridized model based on the combination of FCM, particle swarm optimization (PSO), and the level set method for the segmentation of the brain tumor was investigated by Ali et al. [52]. The PSO algorithm was found to improve the conventional FCM clustering algorithm by selecting the optimal centers of clusters for initial contour determination. Then, the level set methods were introduced for final tumor dissection considering the spatial information. However, the noise and non-homogeneity affect the performance of this method.

Recently, Boulanouar and Lamiche [53] introduced a new hybrid method based on a modified fuzzy bat optimization algorithm (MFBA) and the FCM clustering approach termed MFBAFCM for brain tissue classification. The MFBA algorithm was utilized to obtain the optimal cluster centers, which were subsequently utilized as the adaptive initial seed for the conventional FCM. This hybrid approach addressed the problem of the conventional FCM clustering algorithm, which falls into a local, optimal solution. However, the method is still partially sensitive to noise as well as requiring high computational resources and a post-processing step to refine the extracted tumor regions.

Mishro et al. [54] introduced type-2 adaptive weighted spatial FCM (AWSFCM) to overcome the problems of the conventional FCM clustering method, namely, (i) intensity non-uniformity (INU) artifact and sensitivity to noise, (ii) model trapping in local minima, (iii) the problem of equidistant pixels, and (iv) dependency on initial clustering centroids. The type-2 FCM consisted of three main steps. First, noisy pixel misclassification was reduced by embedding neighboring spatial information in the membership function of FCM. Secondly, the effect of INU artifacts was reduced by the incorporation of adaptive weights into the centroids of clusters. The issue of equidistant pixels was resolved by assigning them to a specified cluster by providing higher weights to the pixel closer to the expected decision boundary. Thirdly, the trapping in local minima was avoided by comparing the value of the fitness function with succeeding iterative stages. The approach to segment brain tissue achieved promising results when tested with healthy brain images, but the method was not tested with images containing lesions that affect the normal tissue intensity and cause high INU artifacts. 

In another novel study of brain tissue segmentation, an integrated method of the hidden Markov random field (HMRF) method with a combination of metaheuristic algorithms based on cuckoo search (CS) and PSO was reported by Pham et al. [55]. The model adopted metaheuristic approaches to specify adaptive parameters to perform balancing between the segmented regions, spatial information, and local intensity. Besides, the HMRF method aims to improve the efficiency of searching solutions in the maximum posteriori estimation. This method utilized spatial information and local intensity to control the INU artifacts and the high level of noise existing in the images. However, the model suffers from two drawbacks: (i) the high computational cost due to the problem in setting the appropriate value of parameters and (ii) the difficulty in converting a large number of parameters, including CS and PSO algorithms.

#### 3.3.3. Deep Learning and Traditional Machine Learning

The third sub-category is the hybridization of deep learning and other traditional machine learning methods such as clustering or classification in the segmentation of brain tissue. This hybridized approach was developed to overcome the limitations of promising deep learning-based methods, and the segmentation results are increasingly aggregated using the machine learning methods in the post-processing stage. Various studies [56,57,58,59,60,61,62,63,64,65] have applied this combination of methods. Kamnitsas et al. [56] proposed a dual 3D-CNN pathway to extract both local and contextual information from the 3D brain tumor images. A fully connected 3D CRFs was used to post-process the soft segmentation and effectively removes false positives. This approach uses a dense training strategy to overcome memory requirements but still has relatively poor inference efficiency and a longer computational time owing to the multi-scale patch-based analysis. Similarly, Zhao et al. [57] proposed a new hybridized model of FCNNs and CRFs for semantic segmentation of brain tumors. This model was trained using the 2D image patches in the following three steps: (i) the training of FCNNs model using image patches, (ii) the training of CRFRNNs with FCNNs parameters using image slices, and (iii) the refining of FCNNs and CRFRNN outcomes using image slices. However, the approach is time-consuming during model training and requires CRFs for further structured outputs.

Likewise, Hu et al. [58] combined the multi-cascade convolutional neural network (MCCNN) and CRFs for sub-region segmentation of brain tumors. The segmentation process involves two steps where a multi-cascade network architecture was proposed to consider local label dependency and exploitation of multi-scale features for coarse segmentation as the first step. Secondly, CRFs were used to maintain spatial contextual information of tumor edges and eliminate false positives for refining segmentation results. The method effectively segmented whole tumors using 2D patches obtained from the Flair, T1c, and T2 modalities with lower computational complexity and fewer training parameters. However, this approach suffers from a sample imbalance issue that could affect the segmentation performance for both tumor cores and enhance tumors as they are smaller in size relative to whole tumors. 

A different approach of intra-tumor segmentation was detailed by Yang et al. [59], where a small kernel two-path convolutional neural network (SK-TPCNN) was combined with RFs. The SK-TPCNN system combined both small and large convolution kernels to promote non-linear mapping ability, which can prevent over-fitting and can extract multi-form features. The extracted features were then subjected to an RF classifier to perform joint optimization, which can reduce feature redundancy, hence improving classification accuracy. The RF classifier successfully incorporated redundant features and voxels of each MR image, which were classified into normal brain tissue and different tumor parts. However, the SK-TPCNN produced an over-segmentation result, requiring more training data and a longer computational time. Moreover, the post-processing step is also required for further segmentation enhancement. 

Ito et al. [60] presented another semi-supervised hybrid method with the combination of expectation-maximization (EM) and DNN for the brain tissue segmentation using a probabilistic method to address the labeling error issue. The EM algorithm was used to determine the true label of the unlabeled image, and the expected label was estimated by applying a special noise to the true label. The combination of the EM algorithm and the DNN model uses a small number of annotated images and a high number of unlabeled images to train the probabilistic model. This method improved the accuracy of small region segmentation even with a limited amount of ground truth samples since unlabeled images were incorporated in the training process. However, the proposed work suffers from high computational cost for the training procedure and poor segmentation results, as training the DNN with exact EM uses imbalanced label datasets. A recent study by Khan et al. [61] presented a cascade method for automatic brain tumor segmentation using IoT-generated images. First, three handcrafted features were extracted and subjected to SVM for binary pixel classification to generate confidence surface modality (CSM). The CSM was then exploited as the prior knowledge to address the dynamic appearance challenge of a brain tumor. Then, the CSM, along with MR images, was incorporated into three novel pathways of the CNN architecture. However, the model showed poor performance on intra-tumor region segmentation as the CSM that resulted from SVM-based pixel classification presented information only for two classes (tumor or non-tumor) instead of providing information on individual intra-tumor regions. 

Another study by Jiang and Guo [62] highlighted the hybrid of a 3D fully CNN based on U-net and CRF for multi-class semantic segmentation of brain tumor and the hippocampus. Firstly, the 3D DNN based on U-net was designed to learn the mapping between image volume and labeling volume considering the early fusion of all MR modalities of the training samples. The learned mappings were then fused and applied to the new batch of samples to jointly determine the tissue marking. Moreover, a fully connected CRF was also proposed as the post-processing step to obtain spatially consistent segmentation results. This method effectively combined multiple predictions of the structure’s prior information and ranking of labels. Nevertheless, the proposed method suffers from two major drawbacks, which are (i) the high computational time for training and testing and (ii) the post-processing requirements for further structured segmentation output.

In a more recent study, the 3D DCNN combined with 3D atrous convolution filters, termed AFPNet, was proposed by Zhou, He and Jia [63] for intra-tumor segmentation. The combination of methods aimed to avoid spatial information loss caused by striding and pooling operations of traditional DCNNs and also to enrich the learning features of brain tumors. The 3D atrous convolution layers were applied at various atrous rates to construct an atrous convolution feature pyramid. Then, a 3D fully connected CRF was adopted as the post-processing step to perform more structural segmentation. Despite the advantages, the approach has some disadvantages, including the limited performance of tiny lesion tissue segmentation. Therefore, it has a relatively low segmentation rate for enhancing and core tumor regions in comparison to complete tumor segmentation. Additionally, it also requires a post-processing step for further segmentation enhancement.

An automated segmentation and tumor severity level classification algorithm was suggested by Mahesh et al. [64] based on PSO for tumor segmentation and meta-classifiers, termed FJODCNN, for severity analysis of gliomas. The model consists of three main steps: Firstly, the segmentation of the core and edema regions was performed using the PSO as a clustering algorithm. Secondly, the features were extracted from these regions, and, finally, the classification was performed using the DCNN and optimally tuned by the fractional Jaya opKtimizer algorithm. However, no qualitative or quantitative results were observed for PSO-based segmentation. 

A unified Incremental DCNN model based on Heterogeneous CNNs (HCNN) and CRF for brain tumor segmentation was proposed by Deng et al. [65]. The steps involved in the method include the following: (i) training of the HCNN using image patches, (ii) training of the CRF-recurrent regression-based neural network (RRNN) using image slices with fixed variables of the HCNN, and (iii) adjustment of the whole network with image slices. Three segmentation models were trained, especially with axial, sagittal, and coronal image patches and slices, and finally combined in a voting fusion technique. 

Table 2 displays the strengths and limitations of each sub-category of hybrid methods.

## 4. Discussion

In general, brain image segmentation methods are categorized as intensity-based, machine learning and hybrid, as summarized in Table 3. These approaches have both collective and progressive manners. The collective aim is to segment: (i) healthy brain tissues, (ii) brain sub-structures, and (iii) tumor and intra-tumor regions. The progressive manner is that the complexity of the method increases. Overall, the findings of this review can be classified into four main areas: (i) the main challenges in segmenting brain structures, (ii) segmentation method trends, (iii) types of brain structures that have been segmented, and (iv) the computation time of brain structure segmentation. Despite recent developments in brain image segmentation methods, several challenges do exist.

### 4.1. Main Challenges in Segmenting Brain Structures

**Figure 3:**
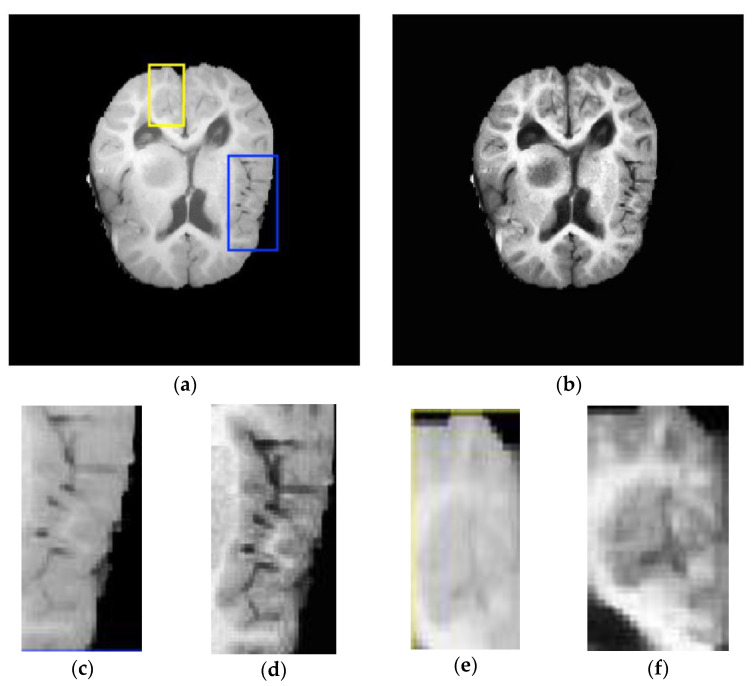
Several challenges (noise, blur effects, and INU artifacts) related to brain segmentation using MR modalities: (**a**) original blur, noisy, and INU slice; (**b**) the resultant slice after the enhancement process; (**c**) original blue line-bounded and magnified region; (**d**) the resultant blue-bounded and magnified region after enhancement process; (**e**) original yellow line-bounded and magnified region; (**f**) the resultant yellow-bounded and magnified region after enhancement process.

This section illustrates some challenges related to brain structure segmentation, especially tumor segmentation, which is the most challenging in MR modalities. The challenges are generally associated with the nature of brain tissue topology and the data acquisition procedure, as illustrated in Figure 3.

These challenges can be summarized as follows:

- Variation in brain tumor shapes: Brain tumors may occur anywhere in the brain tissue and could assume any shape and intensity. This poses difficulty in applying a model based on a shape without prior knowledge or a statistical model to estimate the tumor with a small variance. Besides, the tumor mass affects the arrangement of the surrounding normal tissues, which increases the intensity due to overlap between tumor regions and edema with healthy tissue.
- Intensity inhomogeneity: This is due to the intensity of non-homogeneity of homogeneous tissues during contrast injection and the variations of spatial intensity over each dimension.
- Bias field: The bias field is another challenge faced during the process of brain segmentation in MR images, which is caused by the defects in the acquisition sequences or radiofrequency coil imperfections. The various biases associated with MR images include shading, noise, artifacts, and partial volume effects.
- Non-standardized intensity: The intensity of MR modalities depends on the magnetic fields and radio wave parameters, which are, in turn, influenced by the MR system hardware requirements.
- Data scarcity: Data scarcity is the main weakness of supervised segmentation methods of medical images that leads to overfitting. This implies the model has a good result on the training data but fails to perform well on new data. Mostly, training labels are not available for brain medical image analysis as it requires specialists in this field to label MR images manually, which is a time-consuming process, subjective, and often vulnerable to error.
- Data imbalance: Imbalanced MR image training datasets are one of the main challenges in supervised-based segmentation, especially in the field of brain tumor segmentation or in lesions of the white matter. This is due to the fact that the healthy brain region is greater in region size than the abnormal region. In this case, the training model with imbalanced training datasets often results in an unreliable segmentation biased towards the dominant class with a larger region. For instance, in multimodal MR images, the region of normal brain tissues is larger in size than the abnormal regions that include brain intra-tumor regions. Generally, background and normal brain tissue regions occupy 98.46% of the whole image pixels, while approximately 1.54% of image pixels only belong to the tumor sub-regions. As a solution to tackle this data imbalance issue, several researchers have investigated the data resampling technique. Recently, GAN has been used to synthesize surrogates for the training dataset. This approach provides oversampling of the training dataset with synthetic samples [66,67]. In this work, GAN incorporates structural information of the original dataset to mitigate training data imbalance and scarcity issues. However, these approaches may add redundant data or remove some important details from the original sample. Besides that, a patch-wise sampling approach by Kamnitsas et al. [56] has been adapted to alleviate the data imbalance issue by randomly selecting patches of the normal and abnormal regions from the training datasets. However, this approach suffers the difficulty of determining the right patch size to generate the relevant training data samplings.

### 4.2. Trends in the Segmentation Methods

Various trends in brain structure segmentation approaches using MR images in a semi-automatic and fully automatic mode could be classified into four main classes, namely (i) intensity-based (thresholding and region), (ii) machine learning (SVM, RF, Fuzzy C-mean, CRF, EM, and deep learning), and (iii) hybrid. Intensity-based methods are based on the hypothesis that pixels lying within a specific range belong to one class, despite the simplicity and wide application in the field of brain imaging. The main challenges of traditional intensity-based methods include intensity non-homogeneity and sensitivity to noise for the thresholding-based methods. Meanwhile, the difficulty of incorporating appropriate automated prior knowledge into the energy function to guide the contour evolution is the main constraint of the region-based methods. 

The approaches under the machine learning sub-class depend on manually extracted features such as texture, symmetry, and intensity as input to classifiers for decision making. The segmentation produced by the machine learning approaches suffers from overfitting and loss of contextual information due to the individual classification of each pixel. These approaches are therefore unsuitable for critical applications such as for multi-class brain tumor segmentation as most of the information about a pixel label resides in the neighborhood. Another challenge of handmade features includes significant inter-and intra-user variability during the feature extraction. For instance, the frequently used SVM algorithm is usually adopted for binary classification when the task of tumor segmentation requires the classification of pixels into multiple categories. In contrast, the approaches under clustering-based methods such as FCM and k-means are widely used due to simplicity and applicability. However, being trapped in a local minimum, sensitivity to noise, and INU artifacts are the common drawbacks of these methods. 

Recently, the approaches under the deep learning method, especially CNNs, have outperformed the manually designed features. The segmentation produced by this method can be classified into patch-based and semantic-based methods. In patch-based methods, a small spatial or volumetric patch of an image is selected and subjected to a network. This method partially solves the issue of the imbalanced class label in MR images. However, the approaches under the patch-based model suffer from the time-consuming disadvantage for training and difficulty in determining the patch size. Instead of modeling powerful analysis architecture to categorize numerous image patches, a more elegant network named FCN can be used for semantic-based segmentation using the whole image as an input to the network and trained end-to-end for pixel-wise predictions. The FCN approaches achieved a promising result in the field of brain image segmentation. However, during the process of down-sampling, the U-Net constantly reduces the dimension of the image due to the presence of a pooling layer resulting in poor segmentation accuracy for small tumors that have an overlapping boundary. Furthermore, up-sampling is performed in the original design of the FCN to maintain a low level of spatial features and produce resultant images, which are consistent with the original input size for better image context understanding. However, the up-sampling process cannot completely retrieve the lost information as the U-Net directly connects low-level features to the high-level ones to recover lost information during the down-sampling process, which caused a semantic gap between two layers at the same level. Therefore, the DLA technique was proposed to solve the semantic gap issue using multi-feature refinement and fusion at several levels. The main challenge for implementing CNN-based segmentation is designing an effective network architecture and training strategy. The application of a 2D CNN in a slice-by-slice manner on 3D MR images has a relatively low memory requirement but fails to detect some important spatial information. To address this issue, 3D CNNs with convolutional layers consisting of 3D filters were used to better exploit 3D features. However, this method requires high computational resources. Similar to the 2D CNNs-based segmentation approaches, the 3D CNN architectures were categorized into cascade and UNet. Another group of networks is the 2.5D network, which was generated by subjecting the 2D networks into three orthogonal views. The 2.5D DCNNs approaches achieve a tradeoff between model complexity, memory consumption, and receptive field for 3D DCNN-based methods. Additionally, the 2.5D DCNNs exploit the 2D inter-slice features and have a lower memory consumption than their 3D counterparts. The main issue noted for most of the machine learning and deep learning methods is pixel classification that was conducted without considering the local label dependency of ground truth with the appearance and spatial consistency being ignored. To overcome this issue, the authors integrated deep learning approaches with structuring methods such as CRF; however, the inference can be computationally expensive. Otherwise, cascade architecture can be used to model label dependency by considering the pixel-wise probability outputs where the initial CNN serves as an additional input to subsequent CNNs. These methods can be computationally effective compared to CRF since convolutions are efficient operations. 

Hybrid-based segmentation approaches that integrate two or more different methods within a single system were found to have wide applications in brain image segmentation and demonstrated promising results. Nevertheless, the approach is computationally intensive when running an assemblage of models for both training and inference, which is inappropriate for real-time applications. Specifically, the hybridized approaches with optimization algorithms exhibited an efficient performance in addressing the segmentation challenges and showed promising results in medical image segmentation. Nevertheless, the approach suffers from complexity in setting the optimum value of parameters for computing the fitness function and requires a long computational time. Improved segmentation performance and robust results can be produced with optimization techniques specifically for the hybrid category. Table 3 presents an overview of recent approaches based on different algorithms used for brain tissue segmentation via MR images. A comparative analysis was performed considering the approaches employed, types of segmented tissue, and the type of implementation level. Table 3 also presents a quantitative analysis of various approaches based on different evaluation parameters such as Dice score, Jaccard, accuracy, and precision for brain segmentation. Overall, most of the recent studies focused on multi-class brain tumor segmentation. 

According to Deng et al. [65], the segmentation performance of hybridized deep learning and machine learning methods demonstrated better precision and sensitivity for the BRATS benchmark. Meanwhile, Nema et al. [40] reported better performance using hybrid deep learning-based segmentation for the Dice score. For healthy brain tissue segmentation, the performance of hybrid metaheuristic MOPSO and fuzzy clustering region-based active contours was found to be better than that of the others for both Dice score and sensitivity, as stated by Pham et al. [51]. Boulanouar and Lamiche [53] reported that good results were obtained for both GM and WM segmentation using the Brainweb dataset. For segmentation of the hippocampus structure in the brain, the performance of hybridized deep learning based on 3D U-net with CRF was found to be better than that of the other methods as measured using the Dice score by Jiang and Guo [62]. Nevertheless, increasing the model complexity requires a longer computational time and a larger labeled dataset. In contrast, the combination of the point set registration approach and level set method resulted in a promising result as measured using Dice score as studied by Achuthan and Rajeswari [11]. A reasonable computational time and the use of a labeled dataset without a specific requirement were noted. For grade-wise classification, Khan et al. [6] reported good results when thresholding for segmentation and PART for grade identification were used. However, better performance was observed when metaheuristic (PSO) and thresholding for segmentation and ANN for grade identification were used, as found by Sharif et al. [7]. To the best of our knowledge, there is no approach reported in the literature that could provide the best results for all the parameters being evaluated. Therefore, this review which demonstrates the application of recent segmentation methods, and the pros and cons, will be valuable in guiding future researchers on promising outcomes.

### 4.3. Types of Brain Structure Segmentation

Commonly used image modalities for brain segmentation methods include T1, T2, T1c, FLAIR, CT, 3TMR, and 7TMR modalities. Some of the segmentation methods showed good performance when applied to a single modality, such as brain tissue sub-cortical segmentation, hippocampus, and complete tumor segmentation. However, in the intra-tumor segmentation, multimodality-based approaches performed better. Most of the studies used similar public datasets that are available online such as Brain Multimodal Tumor Image Segmentation Benchmark (BRATS), Brain web, Internet Brain Segmentation Repository (IBSR), and Marmoset, as exhibited in Table 3. The BRATS dataset is widely used in most studies.

### 4.4. Computation Time of Brain Structure Segmentation

Table 3 includes the reported computation time for each of the segmentation approaches. From this review, it is worth noting that the deep learning models that report the longest running time include the training and testing phases. This is due to many factors, such as the level of convolutional layers in the model, spatial size of the filter, number of filters, and other CNN hyperparameters. The run-time of the training phase per subject is approximately three times the computation time of the inference phase. As shown in Table 3, Zhao et al. [57] reported the longest training time among all the reviewed approaches, which took ~12 days for model training. Another work by Ito et al. [60], in which they adapted CRF in the post-processing stage, presented a negative impact on the model training, with a computational time of ~65 h. On the other hand, the longest inference time was reported by Sergio Pereira et al. [21], which took 8 min for the inference phase. In recent years, the rapid development of computer hardware and inference networks with GPU clusters has promoted the application of deep learning-based methods in medical segmentation. However, the extensive hyperparameters settings involved during the network design are still a major bottleneck for clinical application and experimental research.

## 5. Conclusions

This paper presents a critical review of the trending approaches to brain segmentation. The main objective of this review is to provide a better understanding for novice researchers on existing challenges and areas of improvement in brain imaging. The quantitative assessment of the segmentation methods using different evaluation metrics among the various state-of-the-art approaches allows both clinicians and readers to develop new research directions for the accurate diagnosis of brain lesions. The present review suggests that deep learning-based and hybrid-based metaheuristic methods are more efficient for the reliable segmentation of brain tumors. Future recommendations include similar critical reviews for other body organs, such as the knee, stomach, and liver, which can assist researchers in proposing a computer-aided diagnostic framework that will be beneficial for timely cancer diagnosis.

## Figures and Tables

**Table 1:** Strengths and limitations of intensity-based and machine learning approaches for brain segmentation.

Categories	Ref	Strengths	Limitations
Thresholding	[5,6,7,8,9,10]	Simple implementation. Low computation time.	Low performance in heterogeneous regions. Influenced by noise. The setting of the optimal threshold is very subjective. Requires skillful user.
Region based	[11,12,13,14,15]	High segmentation accuracy required for tumor regions. Low computation time. High segmentation efficiency for 3D images. High segmentation performance in complex regions.	Influenced by noise. Requires post-processing step. Requires prior knowledge for parameter initialization.
Traditional machine learning	[16,17,18,19,20]	High segmentation for whole target cases. Simple implementation. Low computation time.	Parameter initialization is subjective. Requires skillful users. Low segmentation performance for semantic type segmentation. Optimum representation features determination is very subjective. Model trapped in a local minimum due to imbalance between exploration and exploitation.
Deep learning	[21,22,23,24,25,26,27,28,29,30,31,32,33,34,35,36,37,38,39,40,41,42,43,44,45]	Adaptive feature map. High performance of semantic-based segmentation. High performance in complex regions. Best segmentation results compared to other categories.	Complex network architecture. Difficult to understand. High computation time. Requires high computational resources.

**Table 2:** Strengths and limitations of hybrid segmentation approaches.

Categories	Approaches	Strengths	Limitations
Contour-based and machine learning	[46,47,48,49]	Provides automatic parameter initialization. Prevents contour-based issues.	The parameter setting is subjective. Requires skillful users.
Metaheuristic and machine learning	[50,51,52,53,54,55]	Optimizes separation features. Provides automatic parameter idealization. Improves response-to-noise ratio. Prevents local minimum to produce optimal results.	Poor performance for local optimization. High system complexity.
Deep learning and clustering or classification	[56,57,58,59,60,61,62,63,64,65]	High performance for intra-tumor segmentation. Encodes spatial information to obtain the further structured output.	Requires high computational time and resources. Needs a post-processing step. Not for real-time applications. Requires a large amount of labeled datasets.

**Table 3:** Summary of brain segmentation approaches (*NA denotes Not Available*).

Approaches Employed	Objectives	Image Modality	Dataset Information	Performance Measure (Accuracy)	Computation Time	Ref
Thresholding	Complete tumor segmentation	MR	Cancer Imaging Archive, 2017	95% (Accuracy)	NA	[5]
Thresholding-based segmentation PART for grade-wise identification	Complete tumor segmentation Grade-wise classification	MR	Self-collected MRI images	95% (Precision)	0.02 s	[6]
PSO and thresholding LBP and deep features extraction GA ANN	Grade-wise classification	NA	RIDER BRATS 2018	99% (Accuracy)	24.90334 s	[7]
ALDE	Healthy brain tissue segmentation	T2	Autism Brain Imaging Data Exchange	NA	1.2202 s	[8]
LSHADE with multilevel thresholding	Complete tumor segmentation	FLAIR	BRATS 2015	0.9172 (Accuracy)	NA	[9]
WCMFO Otsu	Healthy brain tissue segmentation	T2	Harvard medical images	55.834 (PSNR)	2.3496 s	[10]
LACM-BIC k-means Hierarchical centroid shape descriptor	Complete tumor segmentation	T2 T1c	BRATS 2012	91% (DSC)	15.8150 s	[11]
FBB Geodesic level set methods	Complete tumor segmentation	T2 FLAIR	BRATS 2017	89.01% FLAIR 81.59% T2 (True positive rate)	5 min	[12]
Point set registration Level set function	Hippocampus segmentation	T1	OASIS-MICCAI	80.50% (DSC)	NA	[13]
Prior knowledge-based registration Level set function	Hippocampus segmentation	3TMR	ADNI-HarP	84.75% left Hippocampus 73.55% right Hippocampus	NA	[14]
Modified level set	Complete tumor segmentation	T1	BRATS 2015	99% (Average accuracy of 5 images)	NA	[15]
SLIC algorithm RF Multimodal supervoxel	Tumor and intra-tumor segmentation	T1 FLAIR T1c T2	BRATS 2012 BRATS 2013	0.89 whole tumor 0.80 tumor core (Dice Score)	2200 ms	[16]
Multiple classifiers-based collaborative training Feature extraction along with clinical constraints	Tumor and intra-tumor segmentation	T1 FLAIR T1c T2	BRATS 2012 BRATS 2013	0.88 whole tumor 0.81 tumor core 0.74 enhancing tumor (DSC)	NA	[17]
Dominant grey level-based k-means	Healthy brain tissue segmentation	MR	Clinical dataset	Qualitative results	NA	[18]
Multimodal supervoxel Feature selection (ERT) classification Planner voting	Complete tumor segmentation	FLAIR T1c T2	BRATS 2013	0.88 (DSC)	3.5 min prediction time per subject	[19]
Cascade RFs Dense CRF	Tumor and intra-tumor segmentation	T1 FLAIR T1c T2	BRATS 2015 BRATS 2018 ISLES 2015	0.86 whole tumor 0.79 tumor core 0.75 enhancing tumor (DSC)	8 h training 3–4 min inference	[20]
Parallel 2D deep CNN architecture	Tumor and intra-tumor segmentation	T1 FLAIR T1c T2	BRATS 2013 BRATS 2015	88% whole 83% core 77% enhancing tumor (DSC)	8 min testing	[21]
Two-CNN pathway architecture Cascade CNN architecture	Tumor and intra-tumor segmentation	T1 FLAIR T1c T2	BRATS 2013	88% whole tumor 79% tumor core 73% enhancing tumor (DSC)	3 min per epoch training 25 s per slice inference	[22]
Multi-scale CNN	WMH	T1 FLAIR T1IR T2	MRBrainS13	0.87 WM, 0.85 cGM, 0.82 BGT, 0.93 CB, 0.92 BS, 0.93 lvCSF, 0.76 pCSF (DSC)	3–4 min inference	[23]
Dense-Res-Inception Net (DRINet)	Intra-tumor segmentation CSF segmentation in CT images Multi-organ segmentation of abdominal CT images	T1 FLAIR T1c T2 CT	BRATS 2017 Two clinical datasets	83.47% whole tumor 73.21% tumor core 64.98% enhancing tumor 83.42% pancreas 95.96% kidneys 96.57% liver 95.64% spleen (DSC)	21.37 h training 44.46 s inference	[24]
Three modified versions of SEGNET	Tumor and intra-tumor segmentation	T1 FLAIR T1c T2	BRATS 2015	0.87 whole tumor 0.86 tumor core 0.79 enhancing tumor (DSC)	75 ms inference	[25]
Deep cascade neural network	Tumor and intra-tumor segmentation	T1 FLAIR T1c T2	BRATS 2015	89% whole tumor 77% tumor core 77.2% enhancing tumor (DSC)	1.54 s inference	[26]
Combined the symmetric masks in several layers of DCNN	Tumor and intra-tumor segmentation	T1 FLAIR T1c T2	BRATS 2015	85.2% whole tumor 68.1% tumor core 58.1% enhancing tumor (DSC)	9.7 s inference	[27]
Modified cascade 2D U-Net structure	Tumor and intra-tumor segmentation	T1 FLAIR T1c T2	BRATS 2015 BRATS 2017	0.876 whole tumor 0.763 tumor core 0.642 enhancing tumor (DSC)	10 h training 2 min inference	[28]
Deep CNN Cross-modality fusion	Complete tumor segmentation	PET CT T1 T2	Soft-tissue sarcoma (STS)	85% fusing at the feature level 85% fusing at the classifier level 84% fusing at the decision-making level	740 s per epoch training	[29]
Combined two- and three-path CNNs Morphological operation	Tumor and intra-tumor segmentation	T1 FLAIR T1c T2	BRATS 2013	0.86 whole tumor 0.86 tumor core 0.88 enhancing tumor (DSC)	5–7 min inference	[30]
Attention gate residual U-Net model	Tumor and intra-tumor segmentation	T1 FLAIR T1c T2	BRATS 2017 BRATS 2018 BRATS 2019	0.872 whole tumor 0. 808 tumor core 0.80 enhancing tumor (DSC)	NA	[31]
Attention residual U-Net	Tumor and intra-tumor segmentation	T1 FLAIR T1c T2	BRATS 2017 BRATS 2018	87.6% WT, 81.0 TC, 77.3% ET BRATS 2018	NA	[32]
Patch-wise U-net	Healthy brain tissue segmentation	T1	OASIS IBSR	93% in average for CSF, GM, WM OASIS (DSC)	4 h training and inference	[33]
Three-stage cascade FCN	Tumor and intra-tumor segmentation	T1 FLAIR T1c T2		0.8858 whole tumor 0.8297 tumor core 0.7900 enhancing tumor (DSC)	NA	[34]
MRANet	Tumor and intra-tumor segmentation	T1 FLAIR T1c T2	BRATS2015	0.78 whole tumor 0. 68 tumor core 0.60 enhancing tumor (DSC)	2 s inference	[35]
FCNN A battery augmentation	Complete tumor segmentation	FLAIR	MAGNETOM Prisma 3T Siemens	85% average (DSC)	27 ms inference	[36]
R-CNN Chan-Vese level set	Tumor classification and segmentation	T1	Clinical dataset	0.92 (DSC)	NA	[37]
Encoder-decoder based CNN architecture	Tumor and intra-tumor segmentation	T1 FLAIR T1c T2	BRATS 2018	0.8839 whole tumor 0.8154 tumor core 0.7664 enhancing tumor (DSC)	2 days training	[38]
RescueNet Unpaired GAN-based feature learning Scale-invariant	Tumor and intra-tumor segmentation	T1 FLAIR T1c T2	BRATS 2015 BRATS 2017	94.63% whole tumor 85.6% tumor core 93.54% enhancing tumor (DSC)	48 h training 60 s Inference	[39]
3D U-Net architecture	Tumor and intra-tumor segmentation	T1 FLAIR T1c T2	BRATS 2018 Local hospital dataset	0.85 whole tumor 0.77 tumor core 0.67 enhancing tumor (DSC)	NA	[40]
Dense connectivity DCNNs Atrous convolutional feature pyramid	Tumor and intra-tumor segmentation	T1 FLAIR T1c T2	BRATS 2015 BRATS 2017 BRATS 2018	0.8642 whole tumor 0.7738 tumor core 0.7525 enhancing tumor (DSC)	10.616 s inference	[41]
Multi-pathway 3D FCN	Tumor and intra-tumor segmentation	T1 FLAIR T1c T2	BRATS 2018 BRATS 2019	89% whole tumor 78% tumor core 76% enhancing tumor (DSC)	979 s epoch training 86.1s inference	[42]
3D CNN, Dilated convolution	Healthy brain tissue segmentation	T1 FLAIR T1c T2	ADNI MRBrain18 MICCAI 2012	87.2%, WM, 87.2% GM, 89.6% CSF (DSC)	NA	[43]
CNNs test-time augmentation	Tumor and intra-tumor segmentation	T1 FLAIR T1c T2	BRATS 2017 BRATS 2018	87.4% whole tumor 77.5% tumor core 8.3% enhancing tumor (DSC)	NA	[45]
RFs (ccRFs) mpAC	Tumor and intra-tumor segmentation	T1 FLAIR T1c T2	TCGA-GBM TCGA-LGG	90% whole tumor 80% tumor core 73% enhancing tumor (DSC)	7 h training 5 m inference	[46]
Random walks algorithm Weighted averaging algorithm ITRS	Complete tumor segmentation	T1c T2	BRATS 2015	70% HG 70% LG	0.72 s inference	[47]
Otsu k-means	Tumor and intra-tumor segmentation	T2 FLAIR	BRATS 2013	0.8450 enhancing tumor 0.8450 necrotic 0.7834 edema	NA	[48]
Dragonfly algorithm k-Means Level set	Whole tumor	T1 FLAIR T1c T2	BRATS 2017	85.67 (Accuracy)	40–45 s	[49]
PSO-KFECSB	Healthy brain tissue segmentation	T1	IBSR	90.19% (Jaccard Index)	300 s inference	[50]
MOPSO-KFECSB	Healthy brain tissue segmentation	T1	Brain web IBSR	98% CSF 94% GM 96% WM (DSC) 0.9560 (Sensitivity)	4.76 ± 0.15 s per iteration	[51]
Hybrid FCM Particle swarm optimization Level set	Complete tumor segmentation	T1 FLAIR T1c T2	BRATS 2013	93.1% low-grade glioma 89.6% high-grade glioma (DSC)	NA	[52]
MFBAFCM	Healthy brain tissue segmentation	T1	Brainweb	0.9580 CSF 0.9886 GM 0.9833 WM (DSC)	NA	[53]
Type-2 FCM	Healthy brain tissue segmentation	T1 T2	IBSR	0.8381 CSF 0.8381 GM 0.8381 WM (DSC)	9.36 s inference	[54]
HMRF Hybridized of CS and PSO	Healthy brain tissue segmentation	T1	MRBrainS18	0.9374 CSF 0.8744 GM 0.9200 WM (DSC)	900 s inference	[55]
CNN CRF	Tumor and intra-tumor segmentation	T1 FLAIR T1 T2	BRATS 2015 ISLES 2015	0.90 whole tumor 0.75 tumor core 0.73 enhancing Tumor (DSC)	NA	[56]
FCNNs CRF-RNN	Tumor and intra-tumor segmentation	FLAIR T1c T2	BRATS 2013 BRATS 2015 BRATS 2016	0.84 whole tumor 0.73tumor core 0.62 enhancing tumor (DSC) BRATS 2016	~12 d training 2–4 m inference	[57]
Multi-cascaded convolution neural network Fully connected CRFs	Tumor and intra-tumor segmentation	FLAIR T1c T2	BRATS 2013 BRATS 2015 BRATS 2018	88.24% whole tumor 74.81% tumor core 0.7178 enhancing tumor (DSC) BRATS 2018	3 d training 1.5–3 m per image inference	[58]
SK-TPCNN RF classifier Morphological operation	Tumor and intra-tumor segmentation	T1 FLAIR T1c T2	BRATS 2015	0.89 whole tumor 0.80 tumor core 0.87 enhancing Tumor (DSC)	NA	[59]
DNN model using expectation maximization (EM) algorithm	Complete tumor segmentation	T1	IBSR Marmoset	94% (Mean Dice Coefficient)	~65 h training 30 s inference	[60]
SVM Three path CNN	Intra-tumor segmentation	T1 FLAIR T1c T2	BRATS 2015	81% whole tumor, 76% tumor core, 73% enhancing tumor (DSC) BRATS 2013	NA	[61]
3D fully CNN based on U-net CRF	Intra-tumor and hippocampus segmentation	T1 FLAIR T1c T2	BRATS 2017 MNI-HISUB25	88.9% whole tumor 81.3% tumor core 73.49% enhancing tumor (DSC) BRATS2015 90.82 CA1-3 86.65 CA4-DG 95.88% whole MNI-HISUB25	NA	[62]
DCNN 3D atrous 3D CRF	Complete tumor segmentation	T1 FLAIR T1c T2	BRATS 2013 BRATS 2015 BRATS 2018	86% whole tumor 73% tumor core 68% enhancing tumor (DSC) BRATS 2013	7.21 s inference	[63]
PSO FJODCNN	Severity levels of glioma	T1 FLAIR T1c T2	BRATS 2012 BRATS 2018	0.95 (Accuracy)	5.5 s inference	[64]
HCNN CRF-RRNN	Intra-tumor segmentation	T1 FLAIR T1c T2	BRATS 2013 BRATS 2015	96.5 (Precision)	NA	[65]

## References

[1] Siegel R.L., Miller K.D., Jemal A. (2020). Cancer statistics 2020. CA Cancer J. Clin..

[2] Bauer S., Wiest R., Nolte L.-P., Reyes M. (2013). A survey of MRI-based medical image analysis for brain tumor studies. Phys. Med. Biol..

[3] Webster J., Watson R.T. (2002). Analyzing the past to prepare for the future: Writing a literature review. MIS Q..

[4] Moher D., Liberati A., Tetzlaff J., Altman D.G., PRISMA Group (2010). Preferred reporting items for systematic reviews and meta-analyses: The PRISMA statement. Int. J. Surg..

[5] Ilhan A. (2017). Brain tumor segmentation based on a new threshold approach. Procedia Comput. Sci..

[6] Khan S.R., Sikandar M., Almogren A., Din I.U., Guerrieri A., Fortino G. (2020). IoMT-based computational approach for detecting brain tumor. Future Gener. Comput. Syst..

[7] Sharif M., Amin J., Raza M., Yasmin M., Satapathy S.C. (2020). An integrated design of particle swarm optimization (PSO) with fusion of features for detection of brain tumor. Pattern Recognit. Lett..

[8] Tarkhaneh O., Shen H. (2019). An adaptive differential evolution algorithm to optimal multi-level thresholding for MRI brain image segmentation. Expert Syst. Appl..

[9] Aranguren I., Valdivia A., Morales-Castañeda B., Oliva D., Elaziz M.A., Perez-Cisneros M. (2021). Improving the segmentation of magnetic resonance brain images using the LSHADE optimization algorithm. Biomed. Signal Process. Control.

[10] Renugambal A., Selva Bhuvaneswari K. (2020). Image segmentation of brain MR images using Otsu’s based hybrid WCMFO algorithm. Comput. Mater. Contin..

[11] Mbuyamba E.I., Avina-Cervantes J.G., Garcia–Perez A., Romero–Troncoso R.D.J., Aguirre-Ramos H., Cruz–Aceves I., Chalopin C. (2017). Localized active contour model with background intensity compensation applied on automatic MR brain tumor segmentation. Neurocomputing.

[12] Kermi A., Andjouh K., Zidane F. (2018). Fully automated brain tumour segmentation system in 3D-MRI using symmetry analysis of brain and level sets. IET Image Process..

[13] Achuthan A., Rajeswari M. (2019). Segmentation of hippocampus guided by assembled and weighted coherent point drift registration. J. King Saud Univ. Comput. Inf. Sci..

[14] Safavian N., Batouli S.A.H., Oghabian M.A. (2019). An automatic level set method for hippocampus segmentation in MR images. Comput. Methods Biomech. Biomed. Eng. Imaging Vis..

[15] Amarapur B. (2018). Cognition-based MRI brain tumor segmentation technique using modified level set method. Cogn. Technol. Work.

[16] Soltaninejad M., Yang G., Lambrou T., Allinson N., Jones T.L., Barrick T.R., Howe F.A., Ye X. (2018). Supervised learning based multimodal MRI brain tumour segmentation using texture features from supervoxels. Comput. Methods Programs Biomed..

[17] Zhan T., Shen F., Hong X., Wang X., Chen Y., Lu Z., Yang G. (2018). A glioma segmentation method using cotraining and superpixel-based spatial and clinical constraints. IEEE Access.

[18] Nitta G.R., Sravani T., Nitta S., Muthu B. (2019). Dominant gray level-based K-means algorithm for MRI images. Health Technol..

[19] Imtiaz T., Rifat S., Fattah S.A., Wahid K.A. (2019). Automated brain tumor segmentation based on multi-planar superpixel level features extracted from 3D MR images. IEEE Access.

[20] Chen G., Li Q., Shi F., Rekik I., Pan Z. (2020). RFDCR: Automated brain lesion segmentation using cascaded random forests with dense conditional random fields. NeuroImage.

[21] Pereira S., Pinto J.A., Alves V., Silva C. (2016). Brain tumor segmentation using convolutional neural networks in MRI images. IEEE Trans. Med. Imaging.

[22] Havaei M., Davy A., Warde-Farley D., Biard A., Courville A., Bengio Y., Pal C., Jodoin P.-M., Larochelle H. (2017). Brain tumor segmentation with deep neural networks. Med. Image Anal..

[23] Moeskops P., de Bresser J., Kuijf H., Mendrik A.M., Biessels G.J., Pluim J.P., Išgum I. (2018). Evaluation of a deep learning approach for the segmentation of brain tissues and white matter hyperintensities of presumed vascular origin in MRI. NeuroImage Clin..

[24] Chen L., Bentley P., Mori K., Misawa K., Fujiwara M., Rueckert D. (2018). DRINet for medical image segmentation. IEEE Trans. Med. Imaging.

[25] Iqbal S., Ghani M.U., Saba T., Rehman A. (2018). Brain tumor segmentation in multi-spectral MRI using convolutional neural networks (CNN). Microsc. Res. Tech..

[26] Cui S., Mao L., Jiang J., Liu C., Xiong S. (2018). Automatic semantic segmentation of brain gliomas from MRI Images using a deep cascaded neural network. J. Health Eng..

[27] Chen H., Qin Z., Ding Y., Tian L., Qin Z. (2020). Brain tumor segmentation with deep convolutional symmetric neural network. Neurocomputing.

[28] Li H., Li A., Wang M. (2019). A novel end-to-end brain tumor segmentation method using improved fully convolutional networks. Comput. Biol. Med..

[29] Guo Z., Li X., Huang H., Guo N., Li Q. (2019). Deep learning-based image segmentation on multimodal medical imaging. IEEE Trans. Radiat. Plasma Med. Sci..

[30] Sajid S., Hussain S., Sarwar A. (2019). Brain tumor detection and segmentation in MR images using deep learning. Arab. J. Sci. Eng..

[31] Zhang J., Jiang Z., Dong J., Hou Y., Liu B. (2020). Attention gate ResU-Net for automatic MRI brain tumor segmentation. IEEE Access.

[32] Zhang J., Lv X., Zhang H., Liu B. (2020). AResU-Net: Attention residual U-Net for brain tumor segmentation. Symmetry.

[33] Lee B., Yamanakkanavar N., Choi J.Y. (2020). Automatic segmentation of brain MRI using a novel patch-wise U-net deep architecture. PLoS ONE.

[34] Silva C.A., Pinto A., Pereira S., Lopes A. (2021). Multi-stage deep layer aggregation for brain tumor segmentation. Lect. Notes Comput. Sci..

[35] Wu D., Ding Y., Zhang M., Yang Q., Qin Z. (2020). Multi-features refinement and aggregation for medical brain segmentation. IEEE Access.

[36] Lorenzo P.R., Nalepa J., Bobek-Billewicz B., Wawrzyniak P., Mrukwa G., Kawulok M., Ulrych P., Hayball M.P. (2019). Segmenting brain tumors from FLAIR MRI using fully convolutional neural networks. Comput. Methods Programs Biomed..

[37] Gunasekara S.R., Kaldera H.N.T.K., Dissanayake M.B. (2021). A systematic approach for MRI brain tumor localization and segmentation using deep learning and active contouring. J. Health Eng..

[38] Myronenko A. (2019). 3D MRI brain tumor segmentation using autoencoder regularization. International MICCAI Brainlesion Workshop.

[39] Nema S., Dudhane A., Murala S., Naidu S. (2020). RescueNet: An unpaired GAN for brain tumor segmentation. Biomed. Signal Process. Control.

[40] Baid U., Talbar S., Rane S., Gupta S., Thakur M.H., Moiyadi A., Sable N., Akolkar M., Mahajan A. (2020). A novel approach for fully automatic intra-tumor segmentation with 3D U-Net architecture for gliomas. Front. Comput. Neurosci..

[41] Zhou Z., He Z., Shi M., Du J., Chen D. (2020). 3D dense connectivity network with atrous convolutional feature pyramid for brain tumor segmentation in magnetic resonance imaging of human heads. Comput. Biol. Med..

[42] Sun J., Peng Y., Guo Y., Li D., Sun J., Peng Y., Guo Y., Li D. (2021). Segmentation of the multimodal brain tumor image used the multi-pathway architecture method based on 3D FCN. Neurocomputing.

[43] Ramzan F., Khan M.U.G., Iqbal S., Saba T., Rehman A. (2020). Volumetric segmentation of brain regions from MRI scans using 3D convolutional neural networks. IEEE Access.

[44] Milletari F., Ahmadi S.-A., Kroll C., Plate A., Rozanski V., Maiostre J., Levin J., Dietrich O., Ertl-Wagner B., Bötzel K. (2017). Hough-CNN: Deep learning for segmentation of deep brain regions in MRI and ultrasound. Comput. Vis. Image Underst..

[45] Wang G., Li W., Ourselin S., Vercauteren T. (2019). Automatic brain tumor segmentation based on cascaded convolutional neural networks with uncertainty estimation. Front. Comput. Neurosci..

[46] Ma C., Luo G., Wang K. (2018). Concatenated and connected random forests with multiscale patch driven active contour model for automated brain tumor segmentation of MR images. IEEE Trans. Med. Imaging.

[47] Lim K.Y., Mandava R. (2018). A multi-phase semi-automatic approach for multisequence brain tumor image segmentation. Expert Syst. Appl..

[48] Tripathi P., Singh V.K., Trivedi M.C. (2021). Brain tumor segmentation in magnetic resonance imaging using OKM approach. Mater. Today Proc..

[49] Khalil H.A., Darwish S., Ibrahim Y.M., Hassan O.F. (2020). 3D-MRI brain tumor detection model using modified version of level set segmentation based on dragonfly algorithm. Symmetry.

[50] Pham T.X., Siarry P., Oulhadj H. (2018). Integrating fuzzy entropy clustering with an improved PSO for MRI brain image segmentation. Appl. Soft Comput..

[51] Pham T.X., Siarry P., Oulhadj H. (2019). A multi-objective optimization approach for brain MRI segmentation using fuzzy entropy clustering and region-based active contour methods. Magn. Reson. Imaging.

[52] Ali H.A.M., Ahmed M.A.A., Hussein E.M. MRI brain tumour segmentation based on multimodal clustering and level-set method. Proceedings of the 2018 International Conference on Computer, Control, Electrical, and Electronics Engineering (ICCCEEE).

[53] Boulanouar S., Lamiche C. (2020). A new hybrid image segmentation method based on fuzzy c-mean and modified bat algorithm. Int. J. Comput. Digit. Syst..

[54] Mishro P.K., Agrawal S., Panda R., Abraham A. (2020). A novel type-2 fuzzy c-means clustering for brain MR image segmentation. IEEE Trans. Cybern..

[55] Pham T.X., Siarry P., Oulhadj H. (2020). Segmentation of MR brain images through hidden Markov random field and hybrid metaheuristic algorithm. IEEE Trans. Image Process..

[56] Kamnitsas K., Ledig C., Newcombe V., Simpson J.P., Kane A.D., Menon D.K., Rueckert D., Glocker B. (2017). Efficient multi-scale 3D CNN with fully connected CRF for accurate brain lesion segmentation. Med. Image Anal..

[57] Zhao X., Wu Y., Song G., Li Z., Zhang Y., Fan Y. (2018). A deep learning model integrating FCNNs and CRFs for brain tumor segmentation. Med. Image Anal..

[58] Hu K., Gan Q., Zhang Y., Deng S., Xiao F., Huang W., Cao C., Gao X. (2019). Brain tumor segmentation using multi-cascaded convolutional neural networks and conditional random field. IEEE Access.

[59] Yang T., Song J., Li L. (2019). A deep learning model integrating SK-TPCNN and random forests for brain tumor segmentation in MRI. Biocybern. Biomed. Eng..

[60] Ito R., Nakae K., Hata J., Okano H., Ishii S. (2019). Semi-supervised deep learning of brain tissue segmentation. Neural Netw..

[61] Khan H., Shah P.M., Shah M.A., Islam S.U., Rodrigues J. (2020). Cascading handcrafted features and convolutional neural network for IoT-enabled brain tumor segmentation. Comput. Commun..

[62] Jiang H., Guo Y. (2020). Multi-class multimodal semantic segmentation with an improved 3D fully convolutional networks. Neurocomputing.

[63] Zhou Z., He Z., Jia Y. (2020). AFPNet: A 3D fully convolutional neural network with atrous-convolution feature pyramid for brain tumor segmentation via MRI images. Neurocomputing.

[64] Mahesh K.M., Renjit J.A. (2019). Multiclassifier for severity-level categorization of glioma tumors using multimodal magnetic resonance imaging brain images. Int. J. Imaging Syst. Technol..

[65] Deng W., Shi Q., Wang M., Zheng B., Ning N. (2020). Deep learning-based HCNN and CRF-RRNN model for brain tumor segmentation. IEEE Access.

[66] Douzas G., Bacao F. (2018). Effective data generation for imbalanced learning using conditional generative adversarial networks. Expert Syst. Appl..

[67] Salazar A., Vergara L., Safont G. (2021). Generative adversarial networks and Markov random fields for oversampling very small training sets. Expert Syst. Appl..

